# Hsp22 pretreatment protects against LPS-induced hippocampal injury by alleviating neuroinflammation and apoptosis by regulating the NLRP3/Caspase1/IL-1β signaling pathway in mice

**DOI:** 10.18632/aging.204586

**Published:** 2023-03-17

**Authors:** Shengliang Peng, Yun Yu, Juan Li, Danling Jiang, Guohai Xu, Lidong Wu, Jialing Hu

**Affiliations:** 1 Department of Anesthesiology, The Second Affiliated Hospital of Nanchang University, Nanchang, Jiangxi 330006, China; 2 Department of Cardiology, The Second Affiliated Hospital of Nanchang University, Nanchang, Jiangxi 330006, China; 3 Department of Pharmacy, Nanchang University, Nanchang, Jiangxi 330006, China; 4 Department of Ultrasound Medicine, The Second Affiliated Hospital of Nanchang University, Nanchang, Jiangxi 330006, China; 5 Department of Emergency Medicine, The Second Affiliated Hospital of Nanchang University, Nanchang, Jiangxi 330006, China

**Keywords:** neuroinflammation, Hsp22, cognitive impairment, microglia

## Abstract

Neuroinflammation is an important reason for the occurrence and development of cognitive impairment. The Lentiviral vector Hsp22 was constructed for intracerebroventricular injection pretreatment, LPS was used to induce the cognitive impairment model in mice, and the Morris water maze was used to examine the changes in cognitive behavior in mice. LPS was used to induce BV-2 microglial cells, and plasmid pretreatment was used to overexpress Hsp22. HE staining, Nissl staining, immunohistochemistry, immunofluorescence, ELISA and protein blotting were used to examine microglial activation, changes in inflammatory factors, changes in pathway proteins and apoptosis. The results showed that LPS induced microglial expression of NLRP3/Caspase-1/IL-1β signaling pathway protein Iba1, and the inflammatory protein and inflammatory factors IL-1β, IL-6 and TNF-α, the expression of Bax increased significantly, Bcl2 expression decreased, and the learning and memory abilities of mice decreased significantly. Preconditioning with the Hsp22-overexpressing lentivirus attenuated LPS-induced activation of hippocampal microglia, the expression of inflammatory factors and pathway proteins, and apoptosis, and improved cognitive impairment in mice. In addition, plasmid-mediated Hsp22 overexpression reversed LPS-induced inflammation. These findings suggest that Hsp22 overexpression is a promising method for the treatment of cognitive impairment.

## INTRODUCTION

Cognitive impairment is one of the most significant features of many neurodegenerative diseases. With the increases in life expectancy and global surgical patients, Parkinson’s disease (PD), Alzheimer’s disease (AD) and Huntington’s disease (HD), perioperative neurocognitive disorders (PNDs) and other neurodegenerative diseases have become a huge health and economic burdens [1, 2]. Neuroinflammation is a vital factor leading to cognitive impairment and neurodegenerative diseases [3, 4]. To date, the exact pathogenesis of most neurodegenerative diseases (such as AD and PD) is still unclear, and there is a lack of effective treatments. Therefore, there is an urgent need to establish a suitable animal model for further exploration of neuroinflammation-related cognitive impairment and neurodegenerative diseases. Preclinical and clinical research have shown that anti-inflammatory treatments can alleviate the symptoms of cognitive impairment [5, 6].

Lipopolysaccharide (LPS) is a major gram-negative bacterial Toll-like receptor 4(TLR4) ligand. Isolated endotoxin promotes of innate immune response and inflammation, which activates inflammatory cells to produce inflammatory cytokines [7–10]. In addition, injection of LPS into animals can induce neuroinflammation in the hippocampus, leading to cognitive dysfunction [11]. LPS causes anorexia, exploratory behaviors, lethargy, and other phenomena similar to clinically relevant symptoms of human neurodegenerative diseases [12]. Therefore, a growing number of studies have used LPS to activate the inflammatory response and establish models of cognitive dysfunction *in vivo* and *in vitro*, which are effective methods for studying the mechanism of cognitive impairment [13–17]. Although many studies have shown that cognitive impairment is related to a variety of pathological processes, the exact mechanism of these diseases is still not clear.

Microglia are resident macrophages in the brain and the main innate immune cells in the central nervous system (CNS) [18]. Under physiological conditions, constant monitoring of the brain microenvironment plays a key role in neurodevelopment and homeostasis [19, 20]. However, long-lasting pathological stimulation can lead to excessive activation of microglia, leading to neuronal death and the production of pro-inflammatory cytokines (such as tumor necrosis factor (TNF-α) and interleukin-6 (IL-6). Microglia mediate immune responses through pattern recognition receptors, including Toll-like receptors and nod-like receptors (NLRs), among which NLR family pyrin domain-containing-3 (NLRP3) is the most widely studied in the NLRs family [21]. Studies have shown that NLRP3 is involved in neuroinflammation and cognition. This factor plays a vital role in the pathogenesis of the disorders [22, 23]. Importantly, recent research shows that the expression of NLRP3 in the hippocampus of a cognitive impairment model is upregulated, and the impaired function can be reversed by inhibiting the expression of NLRP3 [24–26]. Therefore, targeting the NLRP3-Caspase-1-IL-1β signaling axis may be an important way to improve cognitive impairment, although the exact activation mechanism is unknown.

Heat shock protein 22 (Hsp22), which is also known as H11 kinase, E21G1 and HSPB8, belongs to the superfamily of small heat shock proteins, and has a highly conserved α-crystallin domain [27]. With a molecular weight of 21.6kDa, Hsp22 maintains the integrity of the protein by binding to the hydrophobic region of misfolded and unnatural proteins under stress conditions [28]. In recent years, many studies have revealed that Hsp22 plays a vital role in the regulation of oxidative stress, aging, cancer, apoptosis and autophagy [29–33]. However, although there is increasing interest in the biological role of Hsp22, the role and exact mechanism of Hsp22 in LPS-induced cognitive impairment in the hippocampus remain unclear. Therefore, given that Hsp22 acts as a cytoprotective component in nonneuronal cell lines [34]. It is necessary to clarify the potential mechanism of Hsp22 in neuroinflammation-mediated cognitive impairment and the role of Hsp22 in regulating the activation of the NLRP3/Caspase-1/IL-1β pathway.

To our knowledge, this is the first study to report the key role of Hsp22 in LPS-induced cognitive impairment. Our study showed that Hsp22 could improve cognitive dysfunction by regulating the NLRP3/Caspase-1/IL-1β signaling pathway in hippocampal neuroinflammation induced by LPS. This study helps to further improves our understanding of the molecular mechanism of NLRP3 activation, and provides potential new therapeutic targets for the treatment of cognitive impairment.

## MATERIALS AND METHODS

### Animals and ethics statement

Adult male C57BL/6 mice (7–8 weeks old, 22–26 g) were purchased from Henan Skbeth Laboratory Animal Co., Ltd. (Henan, China). All mice are kept in the Animal Management Center of Nanchang University, the photoperiod was 12 hours, and food and water were freely supplied. The room temperature (RT) was controlled at 23°C ± 2°C, and the relative humidity was maintained at 50% ~ 60%. To reduce the pain of the mice, we obtained the approval of the Institutional Animal Care and Utilization Committee of Nanchang University (Animal Care and Utilization Committee Number: 2016020) before the experimental operation. All experimental procedures were performed in accordance with the guidelines of the International Association for Pain Research.

### Intracerebroventricular injection (IVC)

Briefly, the mice were anesthetized by an intraperitoneal injection (i.p.) of 3% pentobarbital sodium and fixed in a stereotactic frame (RWD Life Sciences, Shenzhen, China). In a sterile surgical environment, skin approximately 1 cm along the midline was cut. Referring to the brain atlas the right ventricle was targeted as follows (millimeters from bregma): 0.8 mm behind the bregma, 1.3 mm on the right side of the median line, and 3.5 mm subdural. The compound (5 μl) was injected at a rate of 1 μl/min with a microsyringe, and the syringe was kept in place for 10 min after instillation to prevent reagent reflux. Gentamicin ointment was applied locally for disinfection. After the operation, the skin was sutured, and the mouse was resuscitated using a thermostatic heating blanket, and then returned to the breeding room for the next experiment.

### Experimental design and establishment of the model

First, we pretreated the mice with the Hsp22 plasmid overexpression. Eighteen mice were randomly divided into the control group, LPS+Lv-Hsp22 group (the lentiviral vector was used to overexpress Hsp22) and LPS+Lv-Hsp22-NC group (the negative lentiviral vector of Hsp22) (*n* = 6 per group). According to the manufacturer’s instructions, the mice in the transfection groups were ICV-injected with Hsp22 (1 × 10^6^ TU). The Hsp22 lentiviral vector was constructed by China Shanghai GeneChem Co., Ltd. After the positioning navigation experiment, the mice in each group were administered saline/Hsp22 respectively. After 72 of hours pretreatment with Hsp22 (refer to Tai for the pretreatment time) [35]. The hippocampus was collected for enzyme-linked immunosorbent assay (ELISA) and Western blot analysis.

Next, mice in the LPS+Lv-Hsp22-NC and LPS+Lv-Hsp22 groups were subjected to LPS stimulation treatment to observe the changes of cognitive behavior and hippocampal neuroinflammation in mice. A total of 12 mice were pretreated with Hsp22 and divided into the LPS+Lv-Hsp22-NC and LPS+Lv-Hsp22 groups (*n* = 6 per group), and 6 mice were randomly selected for LPS stimulation. The administration concentration of LPS was 5 μg, which was dissolved in 5 ul of normal saline (the concentration is 0.9% of Nacl solution), because these doses have been reported to induce disease behavior in mice [36, 37]. As described in the previous protocol, 24 mice were subjected to behavioral tests 1 day before LPS injection. LPS (L2880, O55: B5) was purchased from Sigma-Aldrich (St. Louis, MO, USA).

After the last behavioral experiment, the mice were sacrificed using sodium pentobarbitone (i.p., 45 mg/kg), and hippocampal tissues were collected for hematoxylin and eosin (H&E) staining, Nissl staining, immunohistochemistry, and TUNEL staining. All mice were sacrificed 24 h after the LPS injection, and the collected the hippocampus was collected for protein extraction and Western blot analysis.

### Behavioral tests

The Morris water maze (MWM) was used for cognitive behavioral testing [38]. Testing included one continuous daily of the positioning sailing trial (6 days of training) and one space exploration trial (1 day of training). The MWM device (XR-XM101, Shanghai Xinruan, China) consisted of a circular pool with a diameter of 150 cm and a height of 60 cm. The wall had four water inlet points marked on the top to divide the pool into four quadrants (upper left, upper right, lower right, and lower left), which were filled with tap water (30 cm in-depth, 24–26°C). At the center of the right quadrant, a circular white platform (12 cm in diameter) was placed 3 cm below the water surface. During training, white food additive white pigment (GB25577-2010) was used to dye the pool white and hide the white platform. First, the experimental mouse was placed on the platform for 30 seconds to familiarize it with the surrounding environment. The MWM method includes a continuous daily positioning navigation test and a space exploration test. In the positioning and navigation tests, one of the quadrants was randomly selected and the mouse could travel back to the platform, which was randomly placed in the water, and the mouse was allowed to explore the hidden platform for 60 seconds and stay on it for 15 seconds. If the mouse could not approach the platform within 60 seconds, it was guided to the platform and allowed to stay for another 30 seconds. This process was repeated in the four quadrants, and record the escape latency. At the end of the positioning navigation test, the platform was removed, and the space exploration test was carried out. The number of platform crossings in 60 s, the escape latency and the time spent in the target quadrant were recorded. The MWM test was carried out from 8:00–12:00 in the morning.

### Tissue and serum collection

Twenty-four hours after LPS injection, the mice were deeply anesthetized by an intraperitoneal injection of 1.5% pentobarbital sodium (45 mg/kg), and blood was taken after eyeball removal, and centrifuged at 4000 rpm in a low-temperature centrifuge (Thermo Fisher Scientific, Sorvall Legend Micro 17R). After centrifugation for 15 minutes, the supernatant was used for ELISA analysis, and then the mice were perfused through the heart with cold 0.9% NaCl injection to remove blood cells and proteins in the blood circulation. Next, the brain was removed by craniotomy, and the hippocampal tissue was separated, placed in a cryopreservation tube, and then transferred to a −80°C freezer for Western blot analysis. Finally, PBS (150 mL) was perfused, and 4% polymer was perfused again. Formaldehyde fixation (pH = 7.3, 150 mL), H&E staining, Nissl staining, immunohistochemistry and TUNEL staining were performed on mice.

### Cell culture and groupings

The mouse microglial (BV2) cell line was purchased from Shanghai Tongpai Biotechnology Co., Ltd., China. The culture conditions were 37°C in 95% and 5% CO2, and DMEM high-glucose medium (Cell max, +4500 mg/L glucose) supplemented with 10% fetal bovine serum. During the whole experiment, the cells were randomly divided into the control group and LPS group. Our previous studies showed that 5 ug of LPS at a concentration of 1 μg/ul could induce a significant inflammatory response in microglia [39]. Therefore, we used 5 μg of LPS to stimulate BV2 microglia in this study.

In the second step of the cell transfection experiment, the cells were randomly divided into 2 groups: LPS+OE-Control (Hsp22 empty vector plasmid) and LPS+OE-Hsp22 (Hsp22 overexpression plasmid). Simply put, the BV2 cells were seeded in a 6-well plate and grown to 30–40% confluence before being transfected. According to the manufacturer’s instructions (GenePharma, Suzhou, China), the Hspb8 (65064-1) plasmid empty vector and Hspb8 (65064-1) overexpression plasmid (5 ul dissolved in 195 ul of serum-free medium), and GP-transfect-mate (Lot NO: 200703) reagents (dissolve 25 ul of transfection reagent in 175 ul of serum-free medium) were mixed to prepare 400 μl of working solution, and added to a 6-well plate. The medium was replaced with complete medium 8 hours after transfection. After 72 h of transfection, BV2 cells were stimulated with 5 ug of LPS (1 μg/ul) for 24 h, and BV2 cells were collected for protein separation.

### Enzyme-linked immunosorbent assay (ELISA)

The concentrations of proinflammatory cytokines (TNF-α, IL-1β and IL-6) in the mouse hippocampal tissues were detected by ELISA assays using ELISA kits (CUSABIO, Wuhan, China) according to the manufacture’s protocols.

### Western blot analysis

RIPA lysis buffer, phenylmethylsulfonyl fluoride (PMSF), and protein phosphatase inhibitor were mixed in a ratio of 100:1 and added to hippocampal tissue and BV2 microglia to homogenize and incubated for 30 minutes to lyse the samples. Then, the samples were centrifuged at 4°C for 30 min (15 min/time), with a relative centrifugal force at 12,000 relative centrifugal force (rcf), and the supernatant was collected. The protein concentration was determined by a bicinchoninic acid protein assay kit (TIANGEN, Beijing, China). Protein samples (35–50 μg) were separated protein samples by SDS-polyacrylamide gel electrophoresis (10%), and then transferred them to PVDF membranes (0.22 μm). The membranes were blocked in 5% skim milk at RT for 1 h and incubated with the following primary main antibodies overnight at 4°C: rabbit polyclonal anti-Hsp22 (Abcam, 1:1000, ab151552, USA), rabbit monoclonal anti-NLRP3 (Cell Signaling Technology 1:1000, D4D8T, USA), rabbit monoclonal anti-Caspase-1 (Cell Signaling Technology 1:500, E2Z1C, USA), rabbit monoclonal anti Cleaved caspase-1 (Cell Signaling Technology 1:500, E2G2I, USA), rabbit monoclonal anti-IL-1β (Cell Signaling Technology 1:1000, D4T2D, USA), rabbit monoclonal Cleaved IL-1β (Cell Signaling Technology 1:1000, D3A3Z, USA) and rabbit monoclonal anti-Iba1 (Cell Signaling Technology 1:1000, E404W, USA), rabbit polyclonal anti-Bcl2 (ABclonal 1:1000, A0208, China), rabbit monoclonal anti-Bax (ABclonal 1:1000, A19684, China), rabbit polyclonal anti-TNF-α (Proteintech 1:1000, 17590-1-AP, China), rabbit monoclonal anti-IL-6 (Proteintech 1:1000, 66146-1-Ig, China), and mouse polyclonal β-actin (Proteintech 1:5000, 66009-1-Ig, China). Subsequently, the membranes were incubated with horseradish peroxidase-conjugated secondary antibodies respectively (HRP-conjugated Affinipure goat anti-Mouse IgG, SA00001-1, Goat Anti-Rabbit IgG, SA00001-2, 1:6000, Proteintech, China) for 1 h at RT. Bands were visualized with a Bio-Rad Gel Doc EZ imager (Bio-Rad, USA), and the intensity was analyzed by ImageJ software (NIH Image analysis website: http://rsb.info.nih.gov/ij/). β-Actin served as an internal control. Each experiment was repeated at least three times.

### Cell apoptosis analysis

Apoptosis analysis was performed using a TUNEL kit (Roche, 11684817910, Wuhan, China). Hippocampal tissue was collected after the different treatments and add TdT, dUTP and DAPI staining solutions were added. After being incubated in the dark at 4°C for 20 minutes, the samples were washed once with PBS. Cell apoptosis was observed with a Nikon inverted fluorescence microscope (Nikon Eclipse TI-SR, Tokyo, Japan) and analyzed with ImageJ software.

### H&E staining, Nissl staining and immunohistochemistry

H&E and Nissl staining were used to observe neuronal morphology and damage, and immunohistochemistry was used to detect the activation of Hsp22 and microglia in the hippocampus of mice. The brain tissue was embedded in paraffin, and a 4 μm thick brain tissue sections were prepared using a microtome (RM2016, Leica, Germany). After the sections were deparaffinized and rehydrated with xylene and ethanol, H&E staining, Nissl staining or immunohistochemical staining were performed. Using standard H&E and Nissl staining methods, H&E and toluidine blue staining were performed on the 4 μm sections, respectively. For immunohistochemistry, the sections were placed in 100°C (0.01 M, pH = 6.0) citrate buffer to induce antigen retrieval and then soaked them in 3% hydrogen peroxide solution for 10 minutes at RT to quench the endogenous peroxidase activity. After being blocked with 5% bovine serum albumin, the tissue was incubated with anti-Hsp22 antibodies (Abcam, 1:1000, ab151552, USA) or anti-Iba1 (Cell Signaling Technology 1:1000, E404W, USA) at 4°C overnight. Then, the sections were rinsed 3 times with PBS, incubated with anti-rabbit IgG secondary antibodies (1:2000, Boster, China, Wuhan) at RT for 1 h, dyed in 3,3′-diaminobenzidine solution, and reverse stained with hematoxylin. The slices were visualized and analyzed by an optical microscope (NIKON ECLIPSE CI, Tokyo, Japan) equipped with an imaging system and ImageJ software.

### Statistical analysis

GraphPad Prism 8 software was used for statistical analysis. The data are expressed as the mean ± standard error (SEM). Two groups were compared using Student’s *t* test, and multiple groups were compared using repeated-measures one-way analysis of variance followed by post hoc of Bonferroni test or Dunnett’s test.

### Availability of data

The datasets supporting the conclusions of this study are included in this article.

## RESULTS

### Hsp22 overexpression pretreatment ameliorates LPS-induced cognitive impairment of in mice in the MWM test

**Figure 1:**
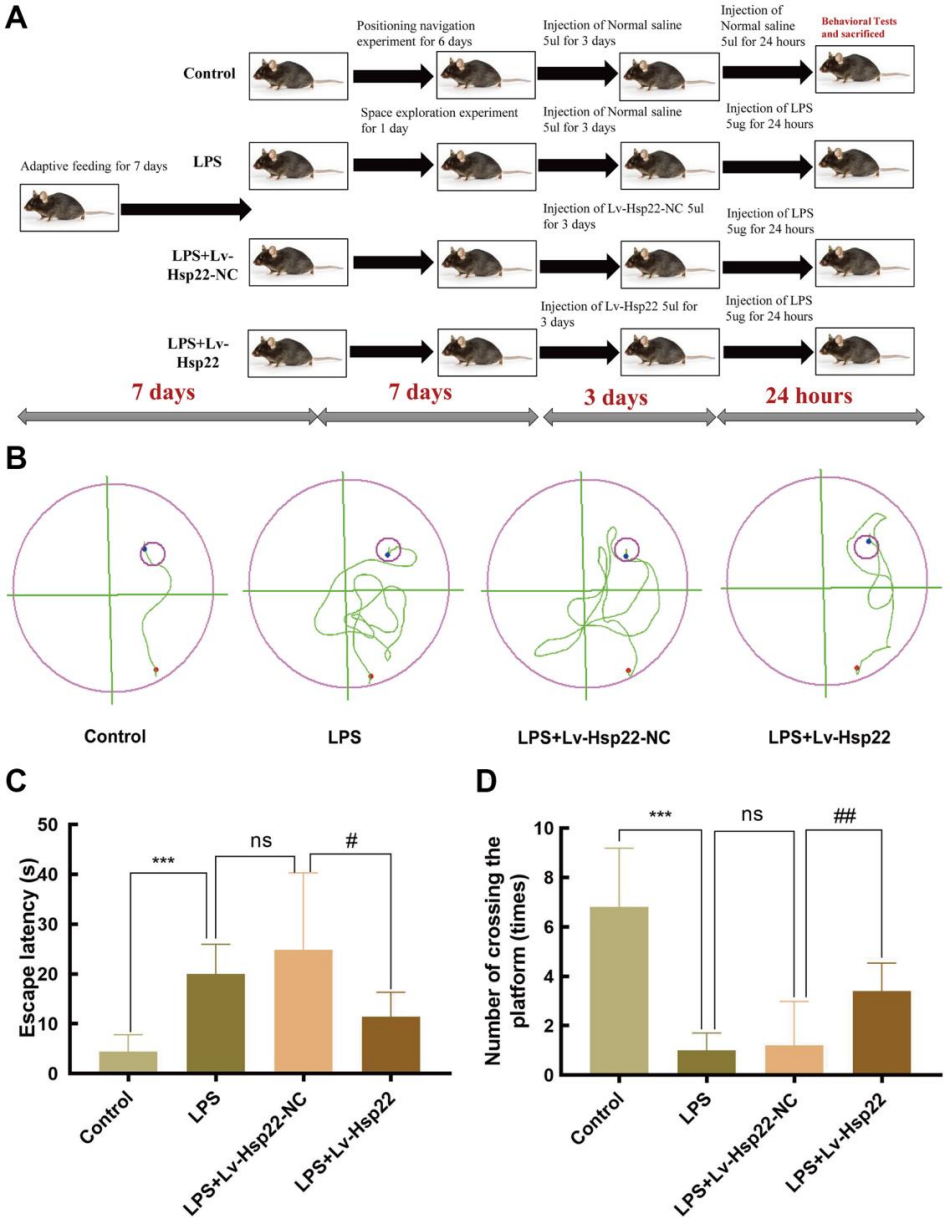
**Hsp22 overexpression preconditioning improved LPS induced learning and memory impairment in mice.** (**A**) Flowchart figure of animal experiment. (**B**) Representative diagram of Morris water maze of mice in each group. Hsp22 overexpression preconditioning reduces escape latency in mouse MWM test (**C**) and Hsp22 overexpression pretreatment increases the number of mice crossing the platform (**D**). (^***^p < 0.005, ^***^p < 0.005 vs. Control; ns: *p* > 0.05, LPS vs. LPS+Lv-Hsp22-NC; ^#^*p* < 0.05, ^##^*p* < 0.01 vs. LPS+Lv-Hsp22-NC).

To study the effect of LPS injection and lentiviral vector-mediated was utilized to Hsp22 overexpression on the learning and memory in mice, the mice were subjected to a MWM experiment after 7 days of adaptive feeding, After MWM training, mice in the LPS+Lv-Hsp22-NC and LPS+Lv-Hsp22 groups were pretreated with the corresponding lentiviral vector for 72 hours, and then stimulated with LPS drugs. Twenty-four hours after LPS injection, the water maze space exploration experiment was performed to observe the changes in the spatial learning and memory abilities of mice before and after the drug was administered. After the experiment, the mice were sacrificed, and serum and the hippocampus were collected for subsequent experiments. No mice in each group died during the experiment. The experiment flow chart is shown in Figure 1A. The water maze activity track was recorded for each group of mice after modeling (Figure 1B). The results of the MWM test showed that compared with the control group, the LPS group and the LPS+Lv-Hsp22-NC group significantly prolonged the escape latencies of mice on day (*p* < 0.05–0.005, Figure 1C), In addition, there was a significant difference in the time spent in the target quadrant between the control group and the LPS-treated group mice (*p* < 0.005, Figure 1D). To further evaluate the effect of Hsp22 overexpression on LPS-induced memory impairment, a lentiviral vector was used to overexpress Hsp22 before LPS injection. The results showed that compared with the LPS group and the LPS+Lv-Hsp22-NC group, the LPS+Lv-Hsp22 group had a shortened escape latency of LPS-treated mice and increased the number of crossing platforms (*p* < 0.01, Figure 1D). These findings indicate that LPS can impair the memory and learning abilities of mice, indicating that the animal model of cognitive impairment was successfully constructed. Hsp22 overpression pretreatment can improve the cognitive decline in mice induced by LPS.

### LPS increases the expression of NLRP3/Caspase-1/IL-1β and proinflammatory cytokines in the hippocampus of mice

**Figure 2:**
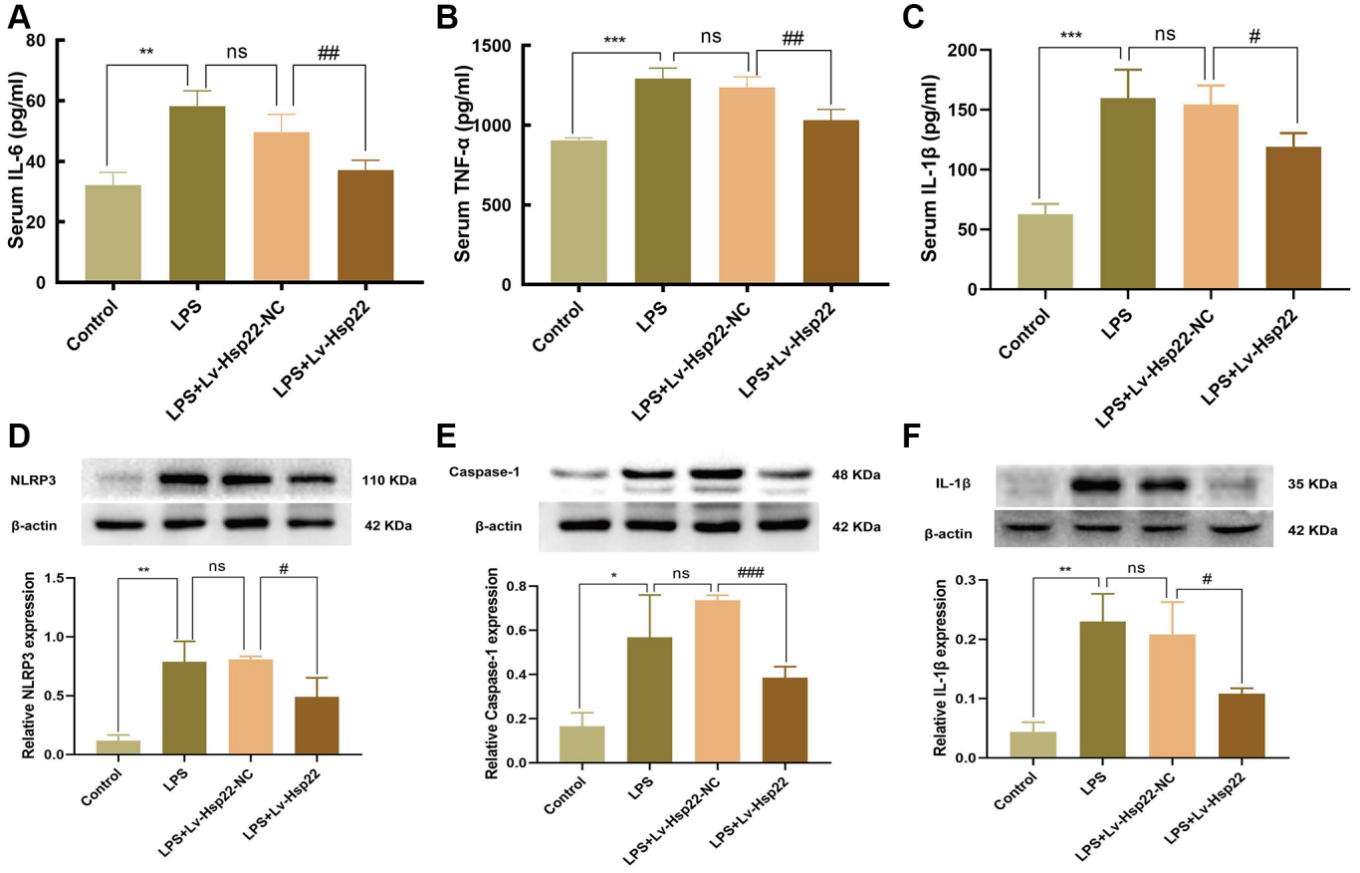
**Effect of Hsp22 on LPS-induced activation of NLRP3/Caspase-1/IL-1β pathways in the mice hippocampus tissue.** The serum levels of IL-6, TNF-α and IL-1β. (**A**–**C**) (^**^p < 0.01, ^***^p < 0.005, ^***^p < 0.005 vs. Control; ns: *p* > 0.05, LPS vs. LPS+Lv-Hsp22-NC; ^##^*p* < 0.01, ^##^*p* < 0.01, ^#^*p* < 0.05 vs. LPS). The protein band of NLRP3, Caspase-1, IL-1β and its expression in the mice Hippocampus tissue (**D**–**F**). (^**^p < 0.01, ^*^p < 0.05, ^**^p < 0.01 vs. Control; ns: *p* > 0.05, LPS vs. LPS+Lv-Hsp22-NC; ^#^*p* < 0.05, ^###^*p* < 0.005, ^#^*p* < 0.05 vs. LPS+Lv-Hsp22-NC).

An increasing number of studies have shown that neuroinflammation is an important factor leading to cognitive impairment [40, 41]. Our previous research showed that the levels of proinflammatory cytokines (IL-6 and TNF-α) in the hippocampus increased within 24 hours of LPS injection [39]. Therefore, we collected blood from mouse eyeballs 24 hours after LPS administration, which was centrifuged, and the obtained serum was used for ELISA analysis. The results showed that compared with that in the control group, the expression of the proinflammatory cytokines IL-6, IL-1β and TNF-α was upregulated in the LPS group and the LPS+Lv-Hsp22-NC group after 24 h. (*p* < 0.01–0.005, Figure 2A–2C). In contrast, the LPS+Lv-Hsp22 group exhibited significantly reduced the expression of these inflammatory factors (*p* < 0.01–0.05, Figure 2A–2C).

The western bolt results (Figure 2D–2F) showed that compared with those in the control group, the protein levels of NLRP3, IL-1β and Caspase-1 in the LPS group and LPS+Lv-Hsp22-NC group were significantly increased (*p* < 0.01–0.05), while, the mice of the LPS+Lv-Hsp22 group had significantly reduced the expression of these proteins in the hippocampus of mice (*p* < 0.05–0.005).

### Hsp22 overexpression decreases pretreatment the expression of NLRP3/Caspase-1/IL-1β and proinflammatory cytokines in the hippocampus of LPS-treated mice

**Figure 3:**
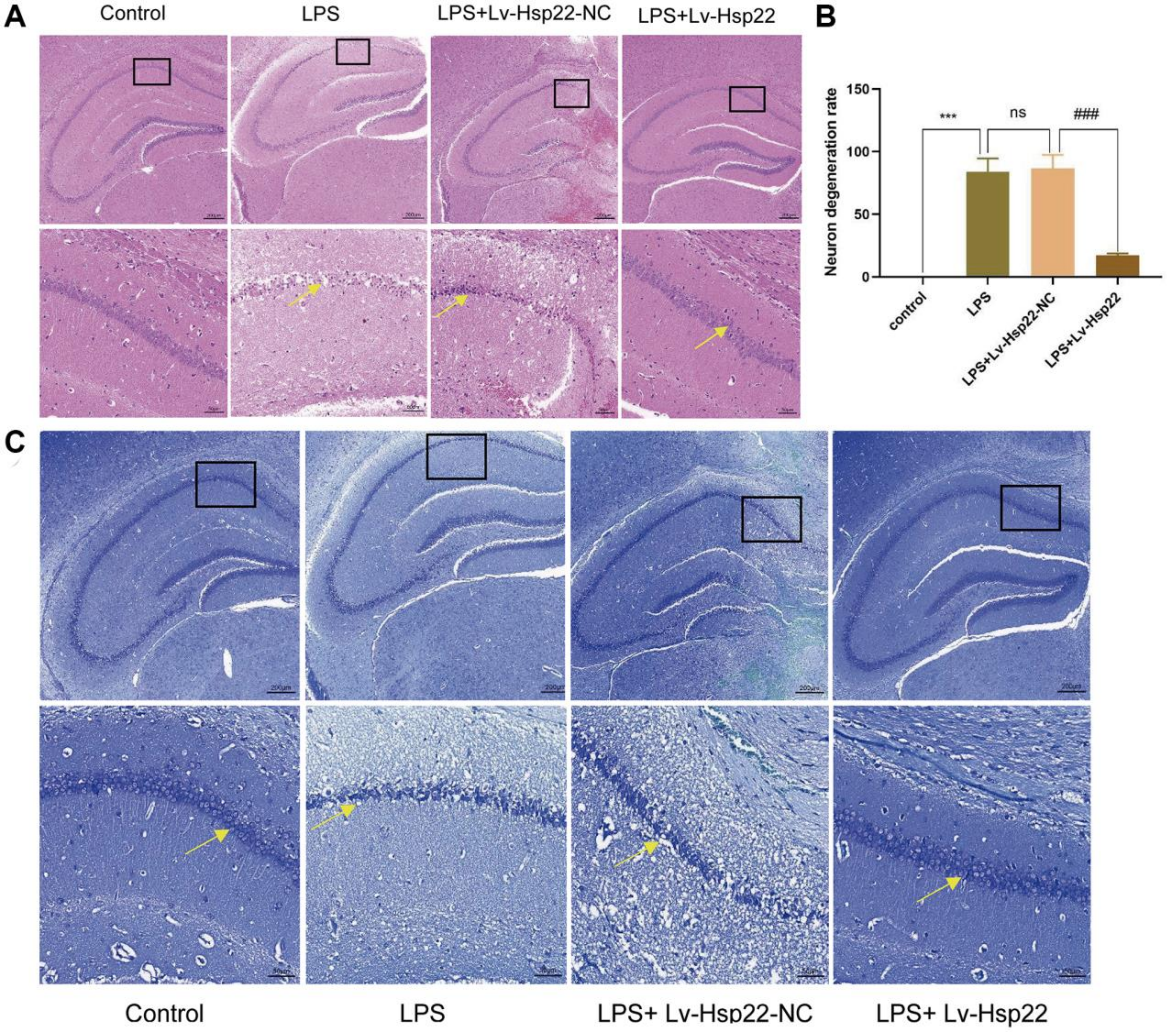
**Hsp22 overexpression pretreatment improves hippocampus tissue damage induced by LPS.** (**A**) Histological analysis of Hippocampus tissue via HE staining (×200). (**B**) The degeneration rate of neurons in LPS group was higher than that in control group, and the difference was statistically significant (^***^p < 0.005). The degeneration rate of neurons in LPS+Lv-Hsp22 group was lower than that in LPS+Lv-Hsp22-NC group, and the difference was statistically significant (^###^*p* < 0.005). (**C**) Histological analysis of Hippocampus tissue via Nissl Staining (×200).

H&E staining was used to observe the neuronal morphology in the hippocampal (Figure 3A, 3B). In the control group, the cells in the hippocampus CA1 area were arranged tightly, with clear nuclei and rich cytoplasms, and there was complete brain parenchymal structure, and no neuronal degeneration or edema. In the LPS group and the LPS+Lv-Hsp22-NC group, a large amount of necrosis in nerve cells and deep staining of nuclear pyknosis were observed in the hippocampal CA1 area, as shown by the yellow arrow; The hippocampal neurons in the LPS+Lv-Hsp22 pretreatment group were arranged more densely and neatly than in the other groups.

Some studies have shown that the structure and function of pyramidal cells in the hippocampus and cortex are related to learning and memory functions [42, 43]. Our histopathological observations on the mouse brain by Nissl-stained mouse brains showed that compared with those in the control group, the pyramidal cells in the hippocampus and cortex in the LPS group and the LPS+Lv-Hsp22-NC group were damaged, as shown by the yellow arrow, which were characterized by Nissl bodies and extensive loss of normal neurons (Figure 3C), However, the number of Nissl bodies in the LPS+Lv-Hsp22 group was decreased. Our Nissl staining results showed that the number of normal neurons in the hippocampus and cortex increased significantly after Hsp22 overexpression was given, suggesting that Hsp22 overexpression has a neuroprotective effect.

**Figure 4:**
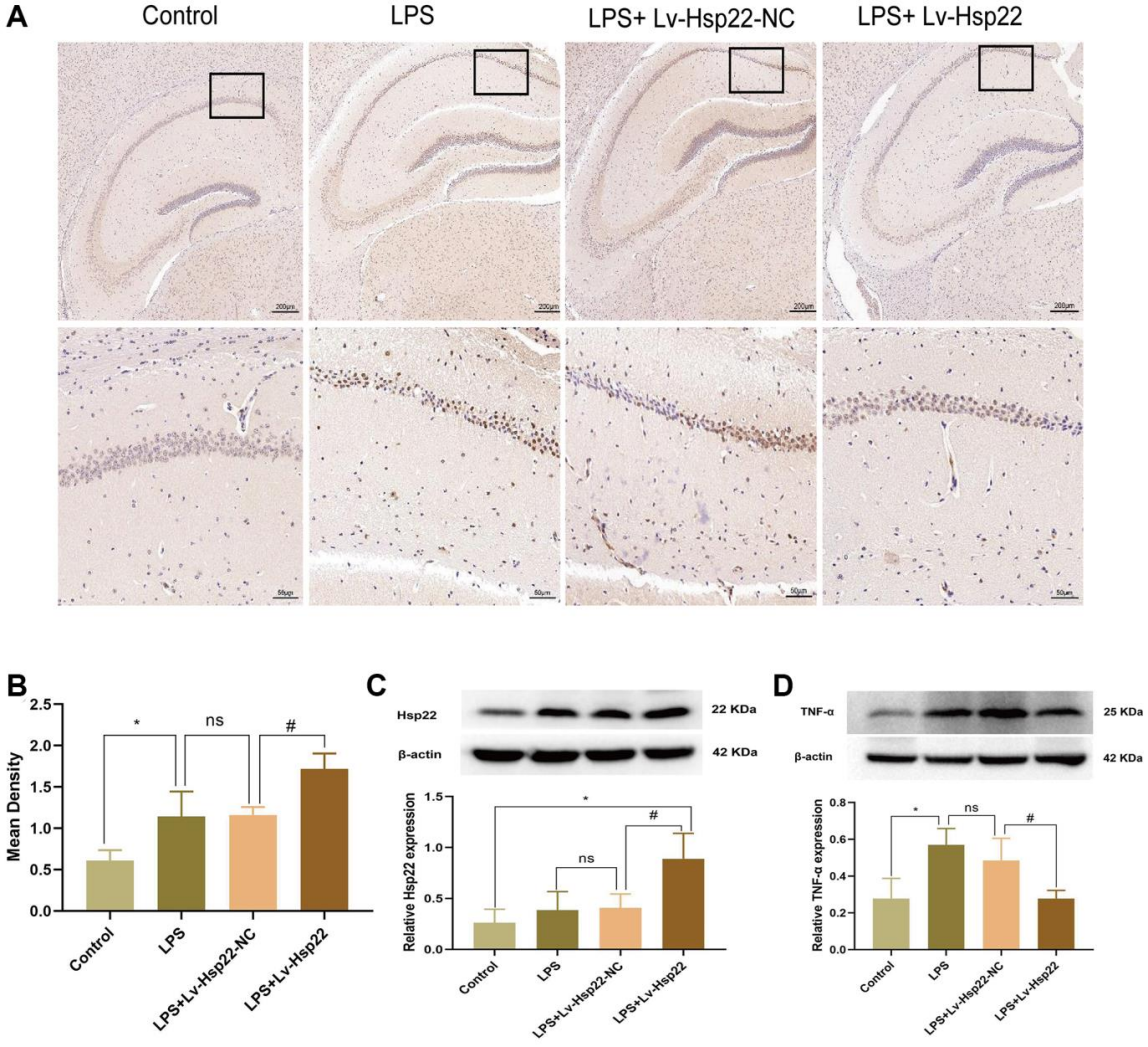
**Hsp22 levels were increased in hippocampus tissue after LPS stimulation and increased expression after Hsp22 pretreatment.** (**A**, **B**) Immunohistochemical staining images of Hsp22(^*^p < 0.05 vs. Control; ns: *p* > 0.05, LPS vs. LPS+Lv-Hsp22-NC; ^#^*p* < 0.05 vs. LPS). (**C**) The protein band of Hsp22 and its expression (^*^p < 0.05 vs. Control; ns: *p* > 0.05, LPS vs. LPS+Lv-Hsp22-NC; ^#^*p* < 0.05 vs. LPS+Lv-Hsp22-NC). (**D**) The protein band of TNF-α and its expression (^*^p < 0.05 vs. Control; ns: *p* > 0.05, LPS vs. LPS+Lv-Hsp22-NC; ^#^*p* < 0.05 vs. LPS+Lv-Hsp22-NC).

Immunohistochemistry showed that the number of Hsp22 positive cells in the hippocampus of mice in the LPS group and the LPS+Lv-Hsp22-NC group was higher than that in the control group. The LPS+Lv-Hsp22 group shown a reduction in these cells (*p* < 0.05, Figure 4A, 4B). Moreover, the Hsp22 protein levels in of the LPS+Lv-Hsp22 group was higher than that in the other groups, indicating that Hsp22 overexpression was successfully established (Figure 4C). These results indicated that Hsp22 overexpression pretreatment can decrease the expression of NLRP3/Caspase-1/IL-1β and proinflammatory cytokines in the hippocampus of LPS-treated mice.

Furthermore, our western bolt results showed that the expression of TNF-α in the LPS group and the LPS+Lv-Hsp22-NC group was higher than that in the other groups, and the LPS+Lv-Hsp22 group had decreased TNF-α expression (*p* < 0.05, Figure 4D).

### Hsp22 overexpression pretreatment decrease the expression of NLRP3/Caspase-1/IL-1β and proinflammatory cytokines in LPS-treated BV2 microglial cells

**Figure 5:**
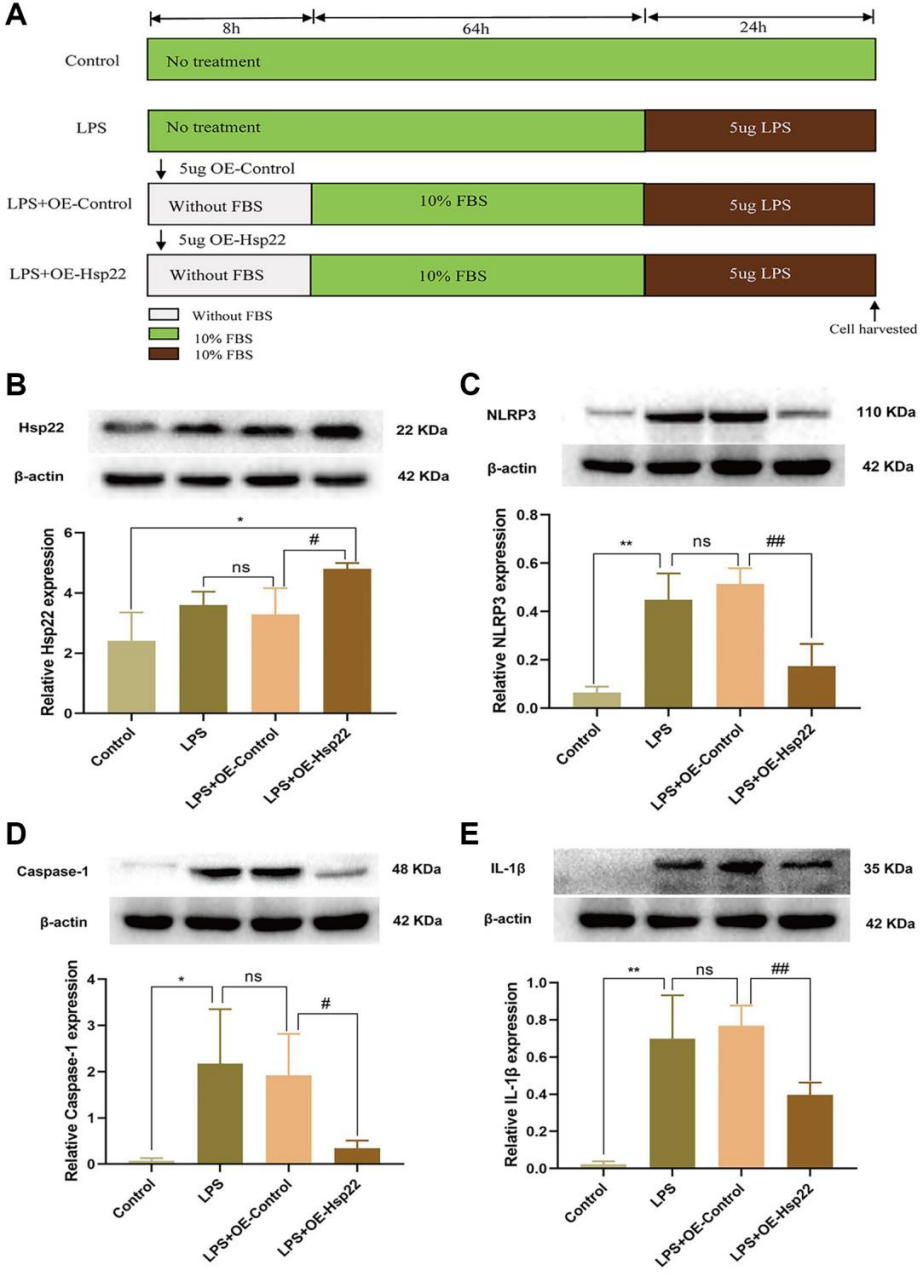
**Effect of Hsp22 on LPS-induced activation of NLRP3/Caspase-1/IL-1β pathways in BV2 Microglial Cells.** (**A**) Cell Modeling Flowchart. (**B**) The protein band of Hsp22 and its expression (^*^p < 0.05 vs. Control; ns: *p* > 0.05, LPS vs. LPS+OE-Control; ^#^*p* < 0.05 vs. LPS+OE-Control). (**C**–**E**) The protein band of NLRP3, Caspase-1, IL-1β and its expression. (^**^p < 0.01, ^*^p < 0.05, ^**^p < 0.01 vs. Control; ns: *p* > 0.05, LPS vs. LPS+OE-Control; ^##^*p* < 0.01, ^#^*p* < 0.05, ^##^*p* < 0.01 vs. LPS+OE-Control).

Next, we used LPS stimulation to study the effect of Hsp22 overexpression preconditioning on BV2 microglia in neuroinflammation *in vitro*. The cell modeling process is shown in Figure 5A. We analysed the morphological change of microglia (Supplementary Figure 1). Western blot analysis showed that the expression of Hsp22 was higher than that in the other groups, indicating that the OE-Hsp22 was successfully transfected into BV2 microglial cells (*p* < 0.05, Figure 5B). Western blotting was used to measure the expression of NLRP3, Caspase-1, and IL-1β. The results showed that the protein expression levels in the LPS group and the LPS+Lv-Hsp22-NC group were higher than those in the control group (*p* < 0.01–0.05, Figure 5C–5E), The protein expression level of cleaved form of caspase-1 and IL-1 β has a significant difference respectively (*p* < 0.05–0.005, Supplementary Figures 2, 3). After overexpressing Hsp22 in BV2 microglia that were treated with LPS, the expression of NLRP3, caspase-1, and IL-1β was significantly reduced compared with LPS group (*p* < 0.01–0.05, Figure 5C–5E). Compared with that in the LPS+Lv-Hsp22-NC group, the difference was not statistically significant (*p* > 0.05, Figure 5C–5E), and the protein expression level of cleaved form of caspase-1 and IL-1 β has a significant difference respectively (*p* < 0.05–0.005, Supplementary Figures 4, 5). In summary, Hsp22 overexpression pretreatment can decrease the expression of NLRP3, Caspase-1, and IL-1β.

### Hsp22 overexpression pretreatment attenuates inflammation in the hippocampus and apoptosis in BV2 microglial cells

**Figure 6:**
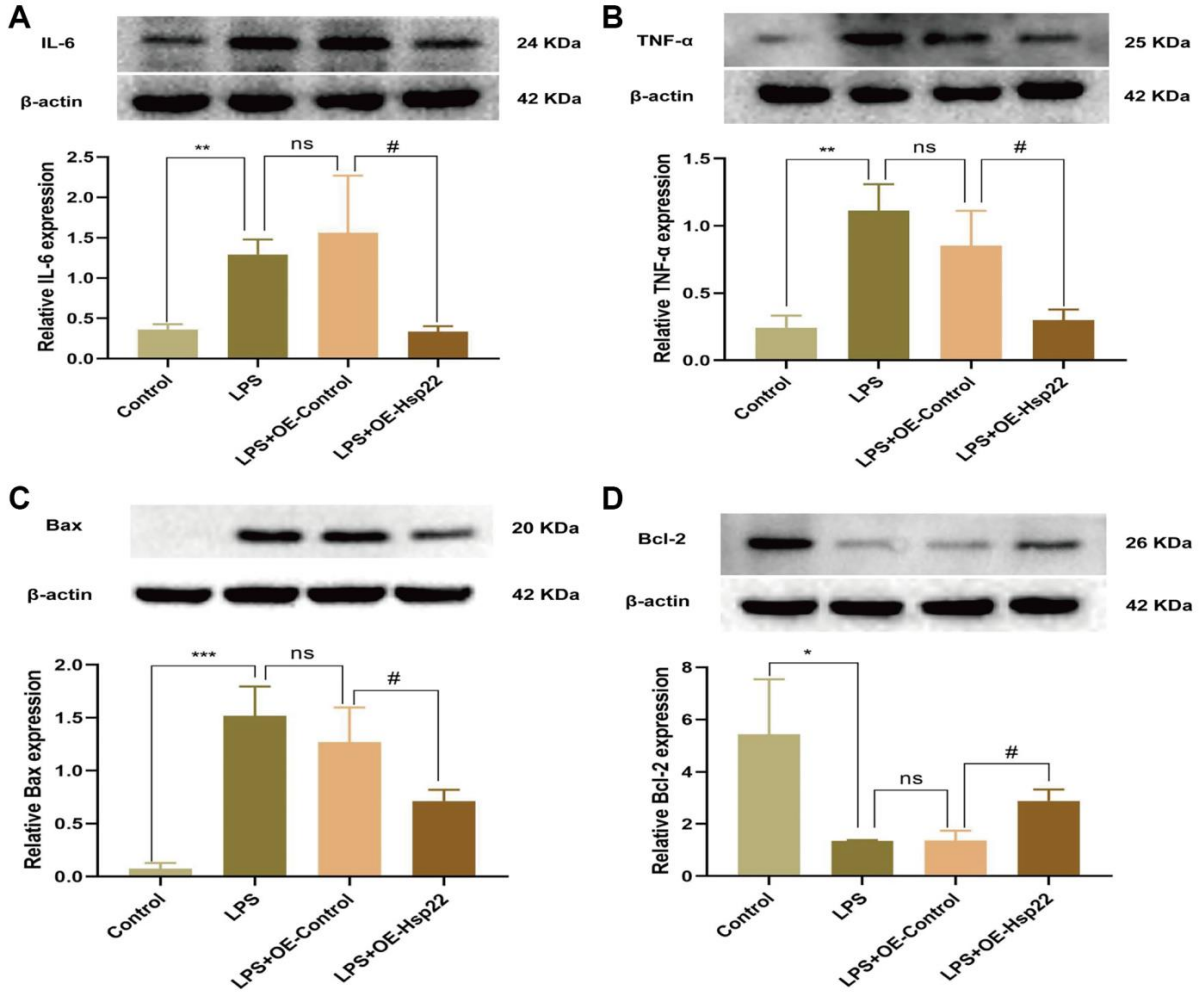
**Hsp22 overexpression pretreatment alleviates BV2 microglial cells inflammation and apoptosis induced by LPS.** The protein band of IL-6 (**A**), TNF-α (**B**) and its expression (^**^p < 0.01, ^**^p < 0.01 vs. Control; ns: *p* > 0.05, LPS vs. LPS+OE-Control; ^#^*p* < 0.05, ^#^*p* < 0.05 vs. LPS+OE-Control). (**C**) The protein band of Bax and its expression (**D**); The protein band of Bcl2 and its expression (***p < 0.005, ^*^p < 0.01 vs. Control; ns: *p* > 0.05, LPS vs. LPS+OE-Control; ^#^*p* < 0.05, ^#^*p* < 0.05 vs. LPS+OE-Control).

Western blotting was used to measure the expression of IL-6 and TNF-α, the results showed that the protein expression levels in the LPS group and the LPS+OE- Control group were higher than those in the control group (*p* < 0.01, Figure 6A, 6B). After Hsp22 overexpression, IL-6 and TNF-α were significantly reduced compared with those in the LPS group (*p* < 0.05, Figure 6A, 6B). We had carried out quantitative real-time polymerase chain reaction (qRT-PCR) experiment and analyzed the mRNA expression level of TNF-α, NLRP3, Caspase-1, IL-1β (Supplementary Figures 6–9), and obtained the results which are similar to Western blotting, which made our results more reliable.

**Figure 7:**
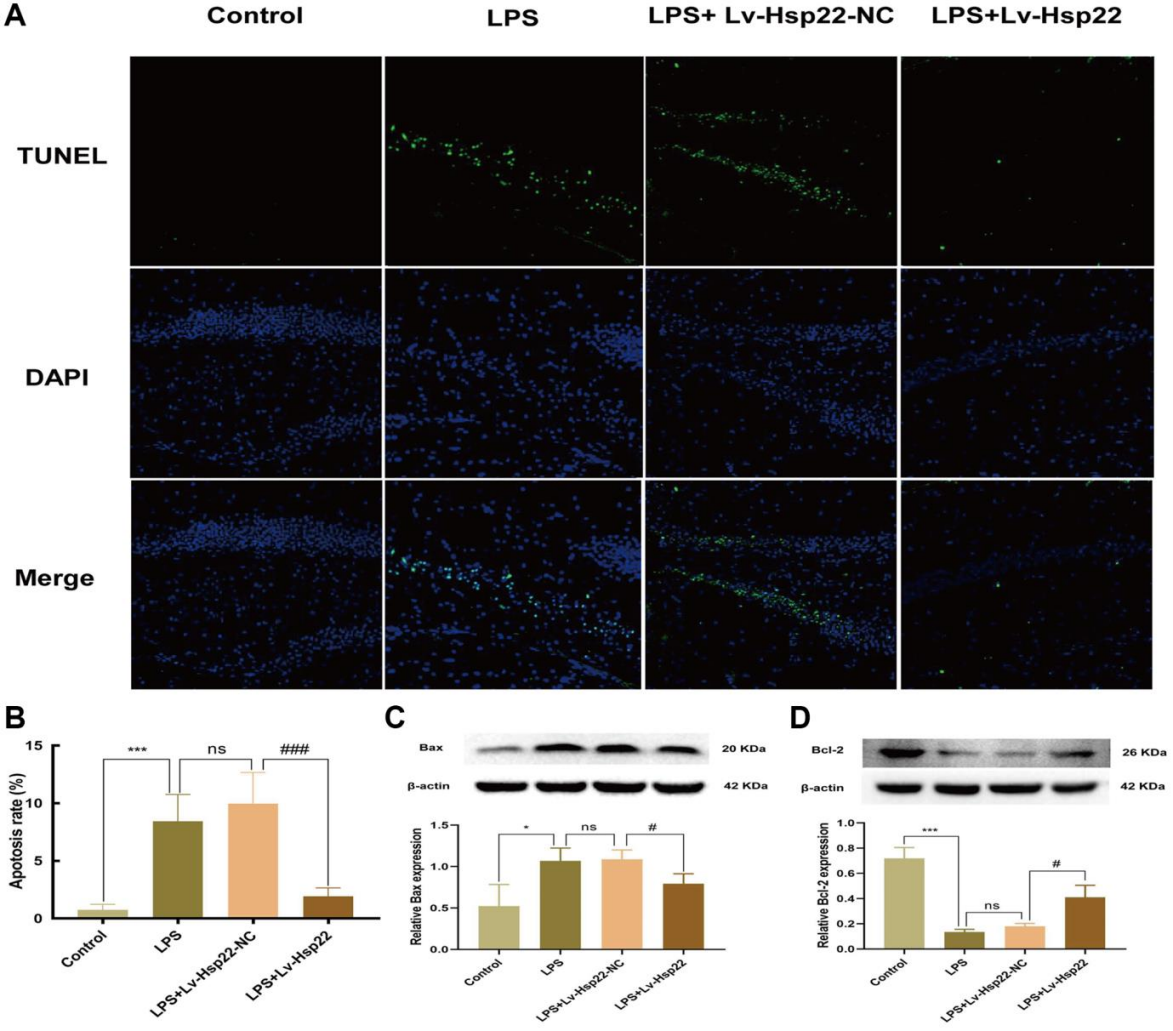
**Hsp22 overexpression pretreatment alleviates hippocampus tissue apoptosis induced by LPS.** (**A**, **B**) Representative images of Tunel-stained brain sections (×200) (^***^p < 0.005 vs. Control; ns: *p* > 0.05, LPS vs. LPS+Lv-Hsp22-NC; ^###^*p* < 0.005 vs. LPS+Lv-Hsp22-NC). (**C**) The protein band of Bax and its expression. (**D**) The protein band of Bcl2 and its expression. (^*^p < 0.05, ^***^p < 0.005 vs. Control; ns: *p* > 0.05, LPS vs. LPS+Lv-Hsp22-NC; ^#^*p* < 0.05, ^#^*p* < 0.05 vs. LPS+Lv-Hsp22-NC).

Apoptosis is a form of the process of inducing programmed cell death that is induced by gene expression to maintain homeostasis under certain physiological and pathological conditions [44]. Previous studies have shown that LPS-induced cognitive impairment is related to apoptosis [17]. The ratio of proapoptotic molecules to antiapoptotic molecules is increased (Bax/Bcl-2). To confirm the influence of inflammation model on apoptosis, we performed TUNEL staining and Western blotting. Western blot analysis of BV2 microglial cells showed that, compared with the control group, Bax was upregulated in the hippocampus in the LPS and LPS+OE-control group, and Bcl2 was downregulated (*p* < 0.05–0.005, Figure 6C, 6D). We analyzed the Bax/Bcl-2 ratio *in vivo* and *in vitro* experiments and the results are the same as reliable (*p* < 0.05–0.005, Supplementary Figures 10, 11). The TUNEL assay and hippocampal tissue protein analysis yielded similar results (*p* < 0.05–0.005, Figure 7A–7D). Hsp22 overexpression in the pretreatment group reversed this outcome. Our research shows that overexpression of Hsp22 may exert a neuroprotective effect by reducing cell apoptosis.

### Hsp22 overexpress pretreatment attenuates microglial activation in the hippocampus of LPS-treated mice and BV2 microglial cells

**Figure 8:**
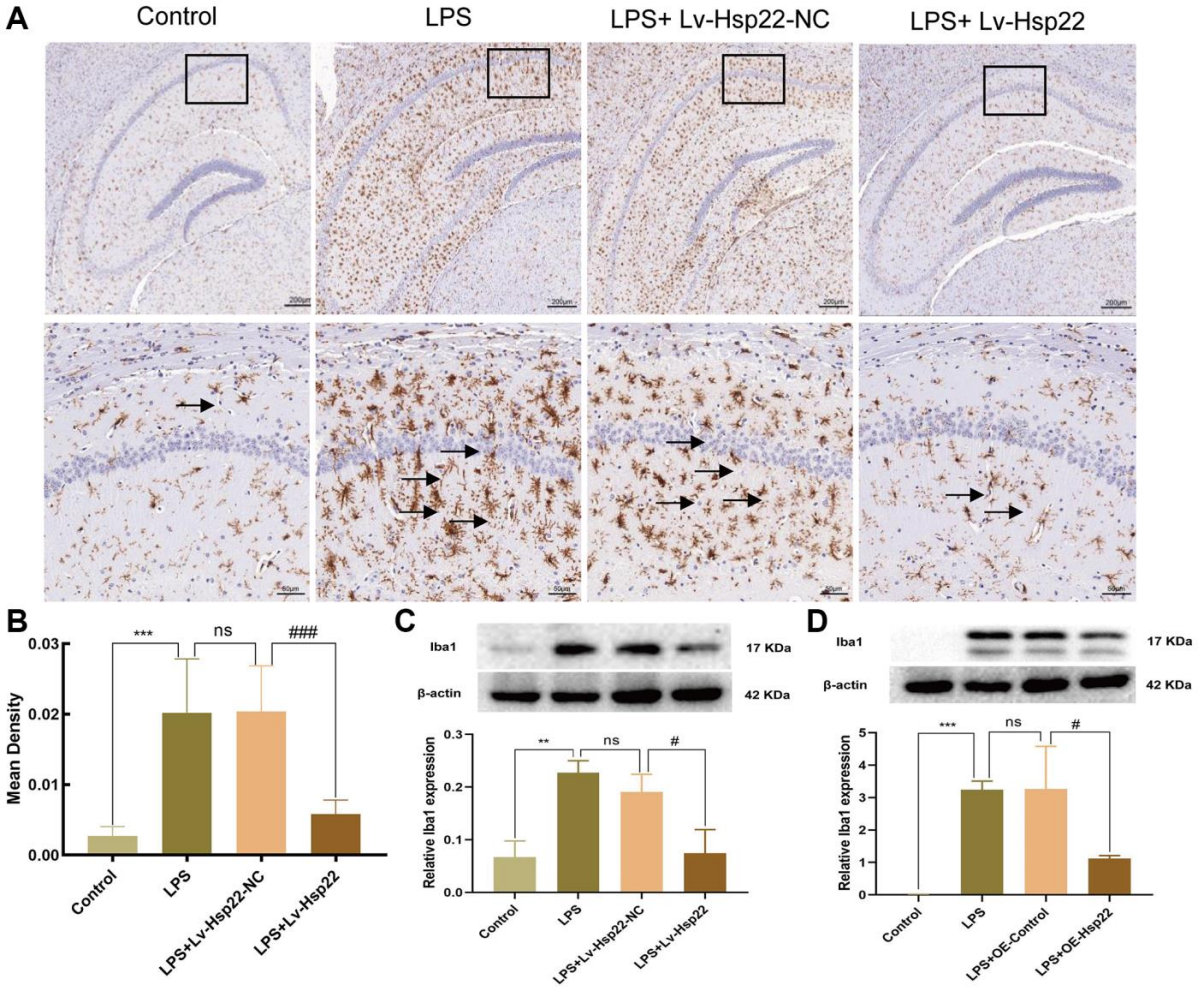
**Hsp22 overexpression pretreatment alleviates Iba1 activation induced by LPS.** (**A**, **B**) Immunohistochemical staining images of Iba1(^***^p < 0.005 vs. Control; ns: *p* > 0.05, LPS vs. LPS+Lv-Hsp22-NC; ^###^*p* < 0.005 vs. LPS+Lv-Hsp22-NC). The arrow points to Iba1 positive cell. (**C**) The protein band of Iba1 and its expression in Hippocampus tissue (^**^p < 0.01 vs. Control; ns: *p* > 0.05, LPS vs. LPS+Lv-Hsp22-NC; ^#^*p* < 0.05 vs. LPS+Lv-Hsp22-NC). (**D**) The protein band of Iba1 and its expression in BV2 Microglial Cells. (^***^p < 0.005 vs. Control; ns: *p* > 0.05, LPS vs. LPS+OE-Control; ^#^*p* < 0.05 vs. LPS+OE-Control).

Iba1 is considered to be an activation marker of microglia [45, 46]. This factor plays an essential role in inflammatory diseases of the central nervous system [47]. In this study, we examined the number of Iba1-positive (microglia marker) cells by immunohistochemistry. We observed that compared with that in the control group, the number of Iba1 positive cells in the LPS group and the LPS+Lv-Hsp22-NC group was significantly increased (*p* < 0.005, Figure 8A, 8B). The Iba1-positive cells in the Hsp22 overexpression pretreatment group were significantly reduced compared with those in the LPS group (*p* < 0.005, Figure 8A, 8B). Next, we also detected the expression of Iba1 protein in the hippocampus and BV2 cells in each group. The expression of Iba1 protein in the hippocampus in the LPS group and the LPS+Lv-Hsp22-NC group was higher than that in the control group. The expression of Iba1 protein in the LPS+Lv-Hsp22 group was lower than that in the LPS group (*p* < 0.01–0.05, Figure 8C). Similar results were obtained in the LPS-induced microglial BV2 cell model *in vitro* (*p* < 0.05–0.005, Figure 8D). These results indicate that Hsp22 overexpression preconditioning negatively regulates microglia activation in a mouse model of cognitive dysfunction.

## DISCUSSION

**Figure 9:**
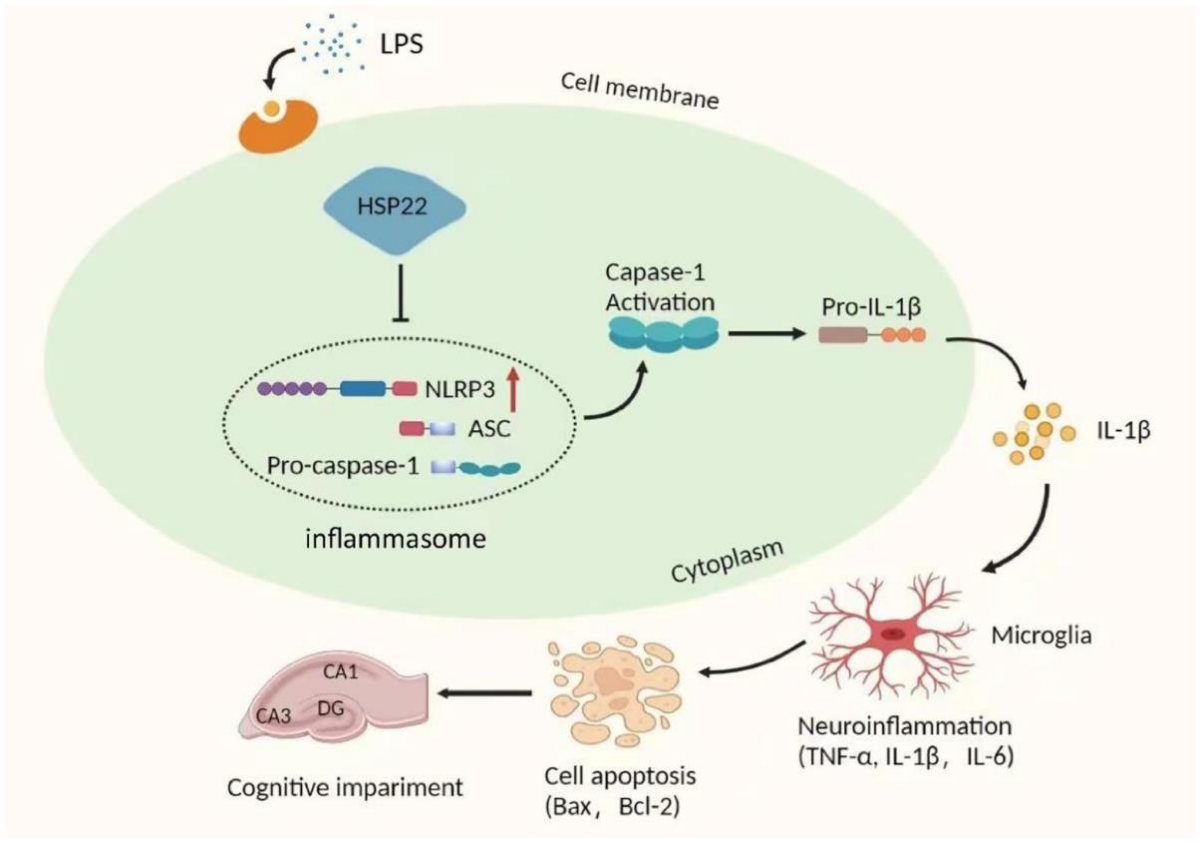
**Schematic depicting the potential mechanism of action of the NLRP3/caspase-1/IL-1β axis in LPS-induced hippocampal neuroinflammation and cognition.** LPS upregulates the expression of Hsp22, which then activates the NLRP3 inflammasome leading to activation of caspase-1, which cleaves pro-IL-1β to IL-1β. As a result, hippocampal neuroinflammation levels are elevated and induce cognitive impairment.

In this study, we explored the potential role of the Hsp22/NLRP3/Caspase-1/IL-1β axis in LPS-induced hippocampal neuroinflammation and cognitive impairment in mice. The related mechanisms were evaluated by overexpressing Hsp22 in the mouse hippocampus and BV2 microglia. The results showed that LPS upregulated the expression of NLRP3/Caspase-1/IL-1β and proinflammatory cytokines (IL-6 and TNF-α), enhanced the hippocampal neuroinflammatory response and activated microglia. In addition, our study shows that pretreatment by overexpressing Hsp22 can improve neuroinflammation and hippocampus-dependent learning and memory decline in LPS-treated mice. In summary, the results show that the activation of the NLRP3/Caspase-1/IL-1β signaling pathway negatively regulates the hippocampal neuroinflammation and cognitive impairment induced by LPS (Figure 9).

Neurodegenerative diseases have complex pathological mechanisms and low cure rates, as well as with chronic and progressive characteristics [48]. Neurodegenerative diseases often affect the quality of life of patients, bring heavy social and economic burdens, and often lead to death. Current treatments include clinical, physical therapy, psychotherapy, and other strategies, with the goal of relieving symptoms or delaying their progression [49]. Cognitive dysfunction is a common clinical manifestation of many neurodegenerative diseases. The underlying mechanism has been extensively explored, but the specific treatment of this syndrome remains to be determined [2]. Increasing evidence shows that neuroinflammation, especially hippocampal neuroinflammation, is the main factor that triggers cognitive dysfunction [3, 50]. Previous studies have shown that LPS-induced cognitive impairment models, such as AD and PD, are effective methods for studying cognitive impairment mechanisms [51, 52]. Published research shows that the MWM test is a robust, reliable and most popular hippocampus-dependent cognitive function test [53]. To this end, thus, we established a neuroinflammation model with LPS to explore cognitive dysfunction, and chose the MWM test to evaluate cognitive function. As expected, the MWM test results showed that after mice were administered LPS by icv injection, significant cognitive decline was observed, suggesting that our LPS-induced cognitive impairment model was successful.

Published studies have shown that the NLRP3 inflammasome triggers the activation of Caspase-1, which cleaves the inactivated cytokine precursors (IL-1β and IL-18) into proinflammatory cytokines (IL-1β and IL-18) [54]. The inflammasome plays a vital role in the activation of CASP1 and the maturation of IL-1β. Previous studies have shown that the NLRP3 inflammasome is associated with many neurodegenerative diseases, such as type 2 diabetes (T2D) and PD [55], and cognitive impairment caused by surgery and anesthesia. Many methods have been identified to inhibit the NLRP3 inflammatory signal at multiple steps [56]. Among them, dexmedetomidine reduces NLRP3-mediated neuroinflammation through the ubiquitin-autophagy pathway and improves perioperative neurocognitive impairment in mice [57]. In addition, microRNA-138-5p has been reported to regulate hippocampal neuroinflammation and cognitive impairment in rats through the NLRP3 signaling pathway [39]. Similarly, our results showed that LPS enhanced the expression of NLRP3/Caspase-1/IL-1β and the proinflammatory cytokines TNF-α and IL-6 in the mouse hippocampus and BV2 microglia. Therefore, our findings suggest that the NLRP3 inflammasome may be a new therapeutic target for cognitive impairment, and that targeting the NLRP3/Caspase-1/IL-1β signaling axis is a promising approach for the treatment of postoperative inflammation.

Previous studies have revealed a similar regulatory relationship between Hsp22 and NLRP3 inflammasomes in cognitive dysfunction-related diseases. It has been reported that in doxorubicin-treated mice, NLRP3 overexpression reduces the protective effect of Hsp22 on cardiac function [58]. Hsp22 has anti-apoptotic activity in melanoma, glioblastoma and breast cancer cells have also been reported successively [59]. Moreover, recent studies have shown that LPS can induce neuronal apoptosis, and the proapoptotic protein Bax and antiapoptotic protein Bcl-2 are involved in regulating apoptosis [60]. Similar to previous research results, our results show that Hsp22 overexpression pretreatment can inhibit the expression of NLRP3, Caspase-1, IL-1β and proinflammatory cytokines in hippocampal neurons, improve cognitive impairment in mice, and improve learning and memory capabilities. In addition, we also noticed that the proapoptotic protein Bax was upregulated and the antiapoptotic protein Bcl-2 was downregulated in LPS-treated mice, and Hsp22 overexpression and preconditioning could reversed this phenomenon. These above results suggest that Hsp22 overexpression and preconditioning protects against on LPS-induced neurotoxicity in the hippocampus of mice, which is characterized by a shorter escape latency, a higher target quadrant time ratio and more platform crossing time [15].

In summary, our study demonstrated for the first time that overexpress Hsp22 overexpression can regulate the NLRP3/Caspase1/IL-1β signaling pathway, ameliorate hippocampal neuroinflammation, and improve cognition by reducing microglial dysfunction induced by LPS in mice. Our research shows that the Hsp22/NLRP3/Caspase1/IL-1β axis may be a potential new target to improve neuroinflammation. Under stress conditions, this pathway mediates mitochondrial homeostasis, oxidative stress and apoptosis, and has cytoprotective activity, thereby accelerating the recovery of nerve function [61]. In addition, other studies have reported that Hsp22 inhibits oxidative stress-induced hippocampal neuronal cell death by regulating mitochondrial pathway and that Hsp22 improves lipopolysaccharide induced myocardial injury [62, 63]. In contrast to existing studies, our study is the first to shows that Hsp22 targets the NLRP3/caspase-1/IL-1β pathway to improve LPS-induced cognitive impairment, that Hsp22 preconditioning reduces the LPS-induced inflammatory response and cell apoptosis and improves learning and memory impairment in mice. and more experimental data are needed to accurately explain the neuroprotective effect of Hsp22, which may help identify new strategies to prevent nerve damage. Hsp22 overexpression may protect against LPS-induced neurodegenerative diseases and cognitive impairment.

Our research has several limitations. First of all, in this study, we only focused on the cognitive function in the early postoperative period, and the long-term cognitive function needs to be further studied. Second, we only studied the effect of Hsp22 on the activation of microglia. Astrocytes and other cells involved in the CNS immune response were not explored. Third, in this study, we analyzed the expression of pro-inflammatory M1 phenotype TNF-α, IL-1 β, and IL-6, but the anti-inflammatory M2 phenotypes such as IL-10, CCL18 and CCL22 have not been analyzed. In the follow-up study, we will improve the detection of IL-10, CCL18 and CCL22. In addition, we will further use electron microscopy to observe the changes in the microstructure of mitochondria in the next study, and analyze the indicators such as cytochrome c to further explore the relationship between Bax and mitochondrial translocation in cell apoptosis. Finally, the surgical process itself, anesthesia and injection stress may affect the expression of Hsp22, hippocampal neuroinflammation and the behavior in mice. However, the current study is a preliminary study. There is no doubt that more experimental data are needed to accurately explain the neuroprotective effect of Hsp22 on cognitive impairment. Subsequent studies will identify the potential effects of these factors on hippocampal inflammation and cognitive behavioral changes.

## CONCLUSIONS

In summary, our study demonstrated for the first time that Hsp22 overexpression preconditioning could regulate the NLRP3/Caspase1/IL-1β signaling pathway, inhibit hippocampal neuroinflammation, and improve cognition by inhibiting microglial activation and dysfunction in LPS-treated mice. Our research shows that the Hsp22/NLRP3/Caspase1/IL-1β axis may be a potential new target to improve neuroinflammation.

## Supplementary Materials

Supplementary Figures
