# Supplementary material for: Hsp22 pretreatment protects against LPS-induced hippocampal injury by alleviating neuroinflammation and apoptosis by regulating the NLRP3/Caspase1/IL-1β signaling pathway in mice
Source: Aging (Albany NY). 2023 Mar 17;15(6):1977–2004. doi: 10.18632/aging.204586 (PMC10085591; doi:10.18632/aging.204586)
Supplement: Supplementary Figures [file aging-15-204586-s001.pdf]

SUPPLEMENTARY FIGURES

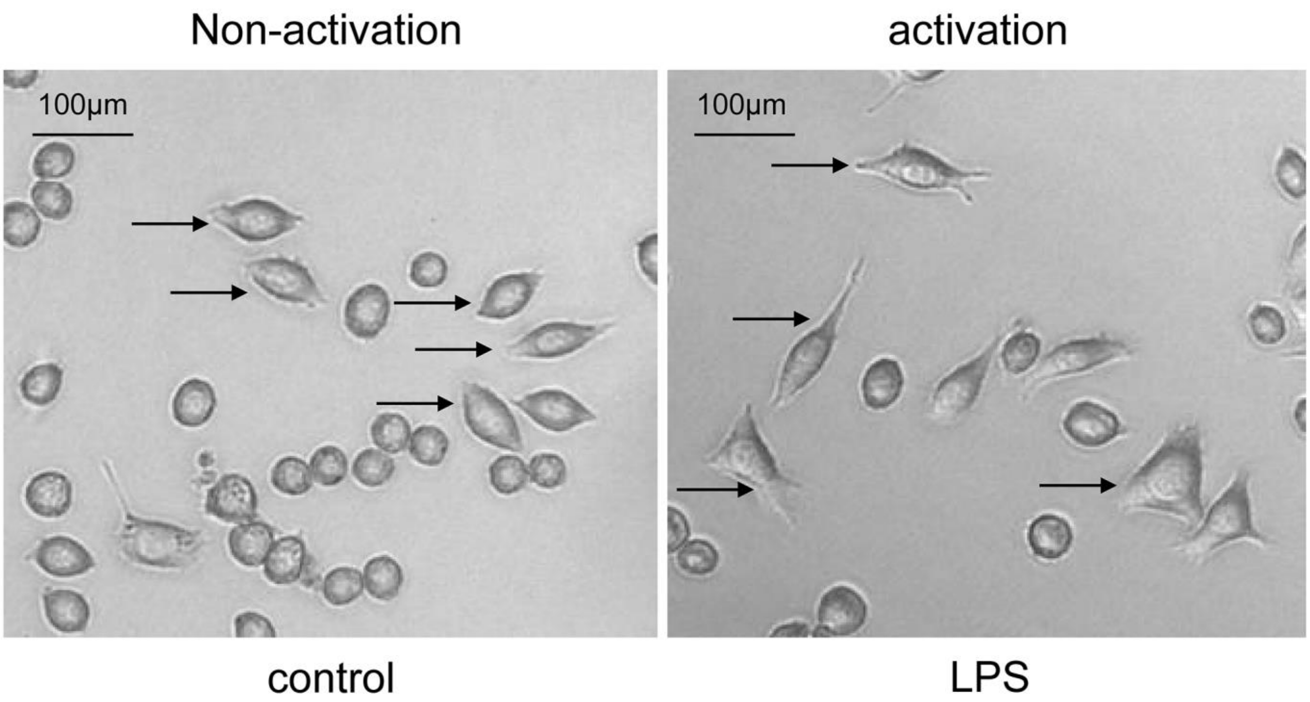

Supplementary Figure 1. The morphological change of microglia. The following microglia can be seen under fluorescent microscope (Olympus, Japan): The cell body of microglia in resting state is small and oval, as shown by the black arrow in the left of Figure S9. LPS is used to induce microglia, and the activated microglia cell body increases and the process becomes shorter, as shown in the right of Supplementary Figure 9.

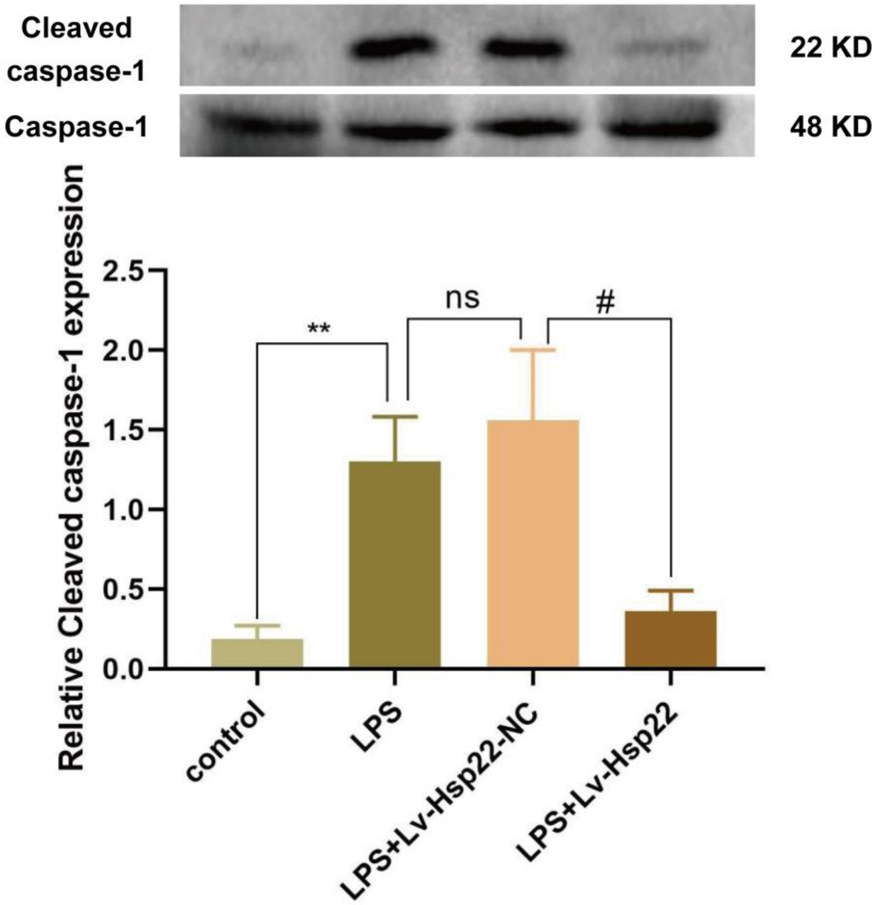

Supplementary Figure 2. The protein expression level of Cleaved caspase-1. Supplementary Figure 1 The protein expression level of Cleaved caspase-1 in LPS group was higher than that in control group, and the difference was statistically significant ($^{**}p < 0.005$). The protein expression level of Cleaved caspase-1 in LPS+Lv-Hsp22 group was lower than that in LPS+Lv-Hsp22-NC group, and the difference was statistically significant ($^{\#}p < 0.05$).

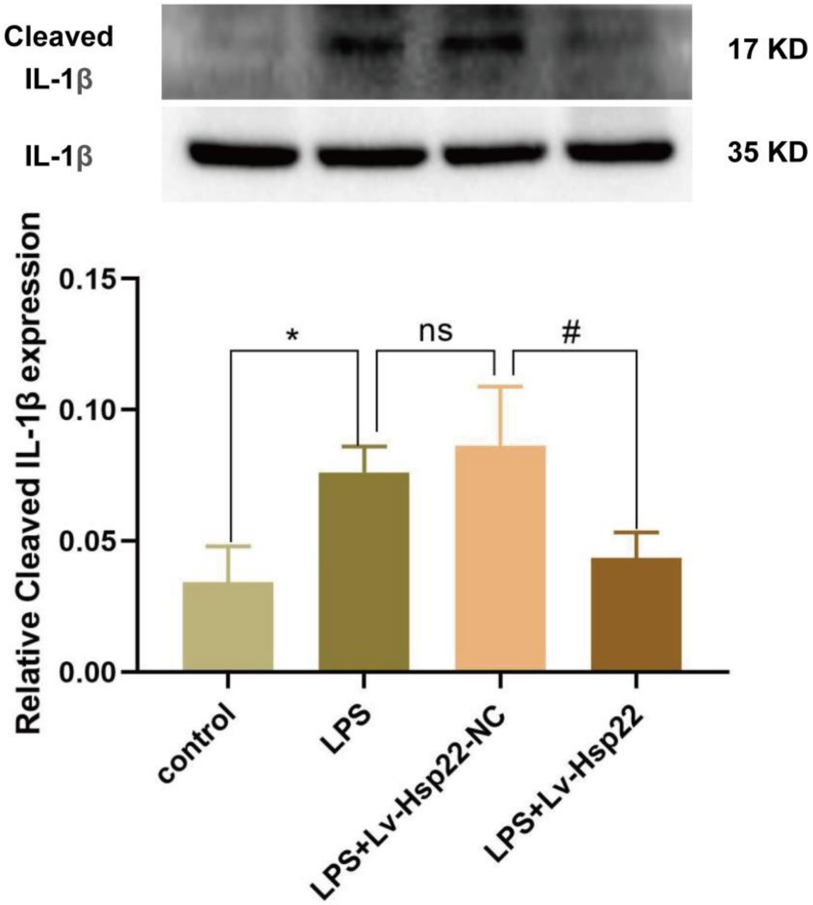

Supplementary Figure 3. The protein expression level of Cleaved IL-1β. The protein expression level of Cleaved IL-1β in LPS group was higher than that in control group, and the difference was statistically significant ($^*p < 0.05$). The protein expression level of Cleaved caspase-1 in LPS+Lv-Hsp22 group was lower than that in LPS+Lv-Hsp22-NC group, and the difference was statistically significant ($^\#p < 0.05$).

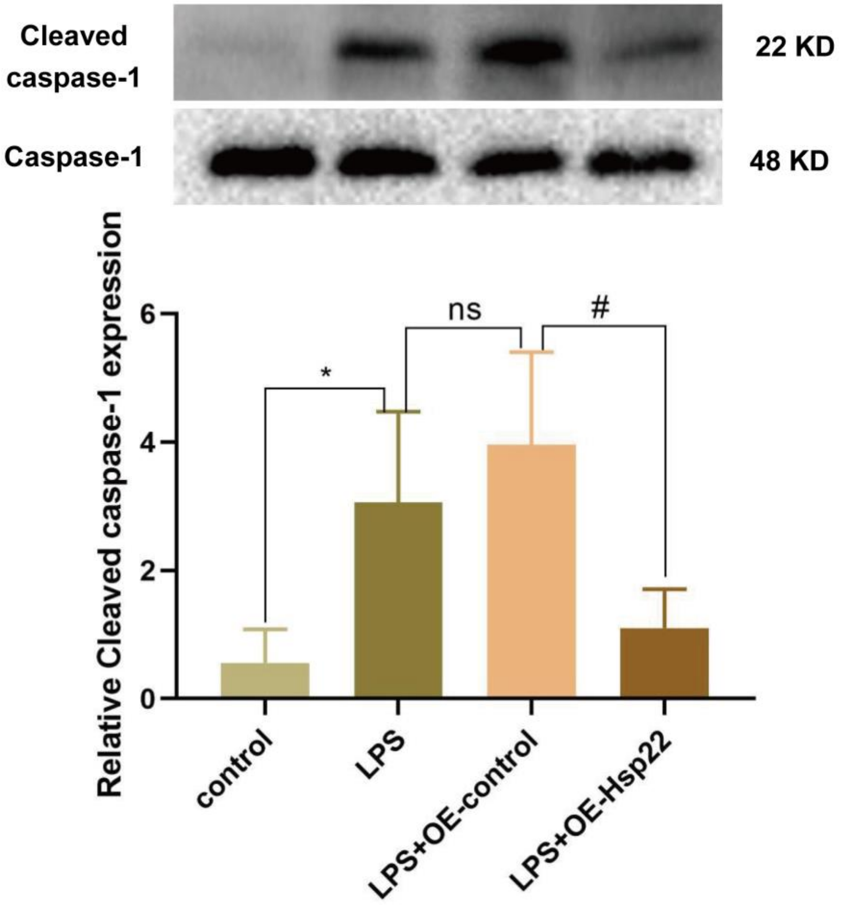

Supplementary Figure 4. The protein expression level of Cleaved caspase-1. The protein expression level of Cleaved caspase-1 in LPS group was higher than that in control group, and the difference was statistically significant ($^{*}p < 0.05$). The protein expression level of Cleaved caspase-1 in LPS+OE-Hsp22 group was lower than that in LPS+ OE-control group, and the difference was statistically significant ($^{\#}p < 0.05$).

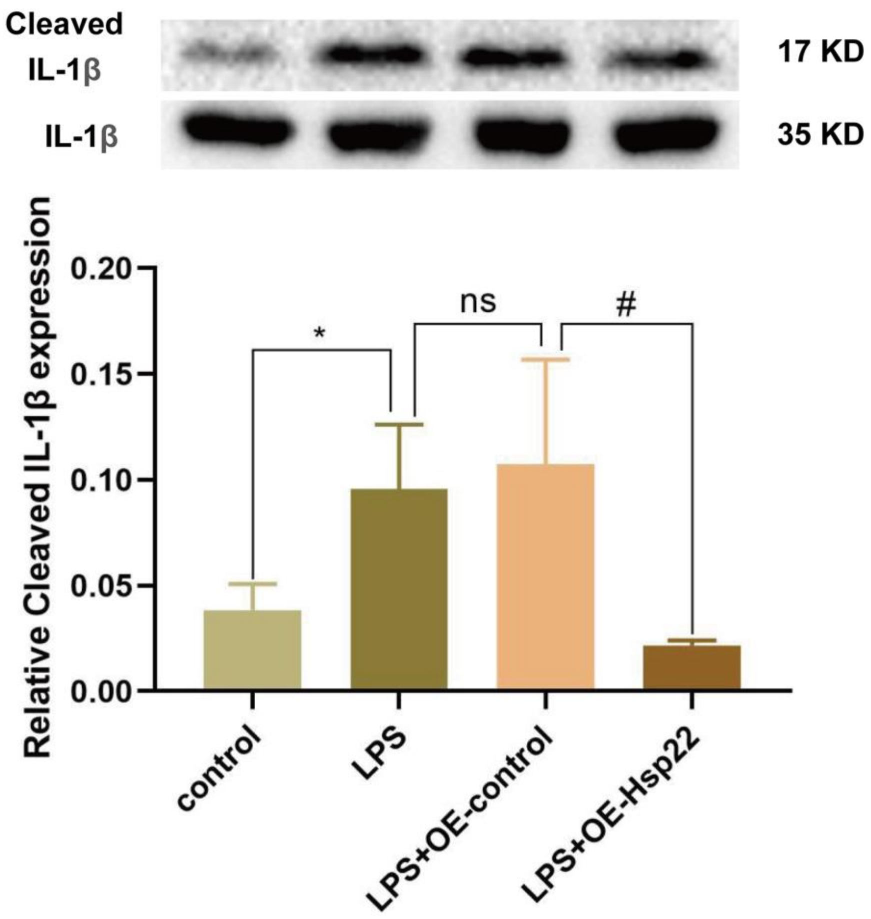

Supplementary Figure 5. The protein expression level of Cleaved IL-1β. The protein expression level of Cleaved IL-1β in LPS group was higher than that in control group, and the difference was statistically significant ($^{*}p < 0.05$). The protein expression level of Cleaved IL-1β in LPS+OE-Hsp22 group was lower than that in LPS+ OE-control group, and the difference was statistically significant ($^{\#}p < 0.05$).

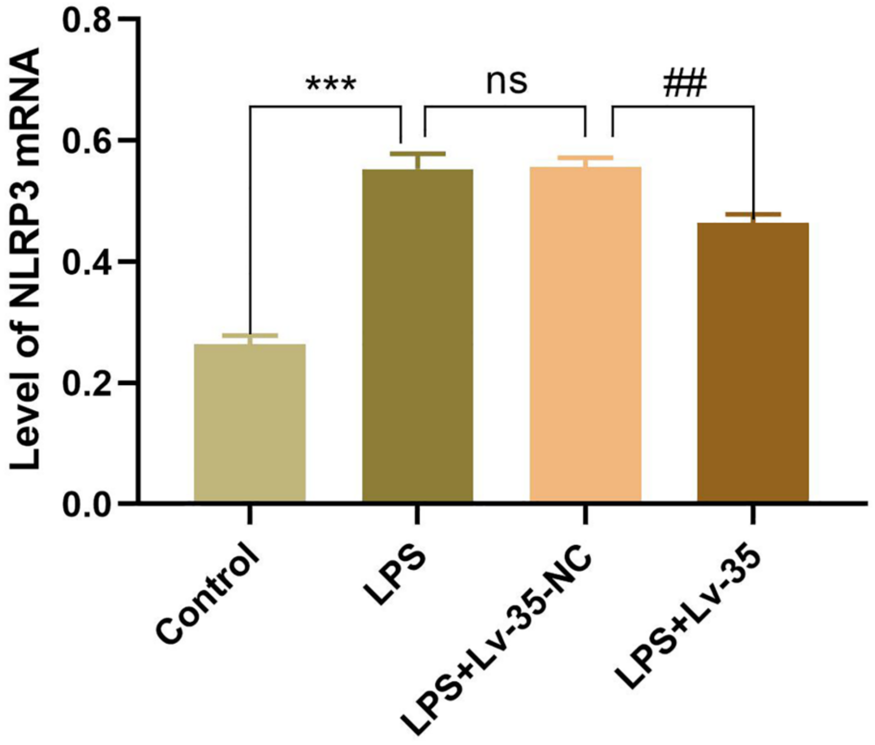

Supplementary Figure 6. The mRNA expression level of TNF-α. The mRNA expression level of TNF-α in LPS group was higher than that in control group, and the difference was statistically significant ($^{**}p < 0.005$). The mRNA expression level of TNF-α in LPS+Lv-Hsp22 group was lower than that in LPS+Lv-Hsp22-NC group, and the difference was statistically significant ($^{\#}p < 0.05$).

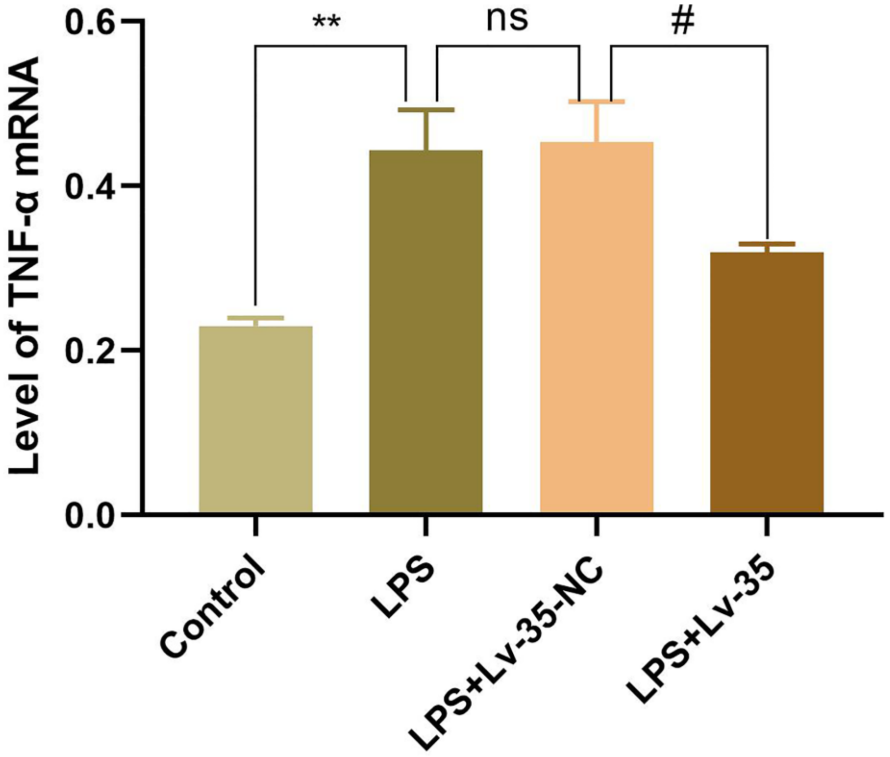

Supplementary Figure 7. The mRNA expression level of NLRP3. The mRNA expression level of NLRP3 in LPS group was higher than that in control group, and the difference was statistically significant ($^{***}p < 0.005$). The mRNA expression level of NLRP3 in LPS+Lv-Hsp22 group was lower than that in LPS+Lv-Hsp22-NC group, and the difference was statistically significant ($^{\#\#}p < 0.005$).

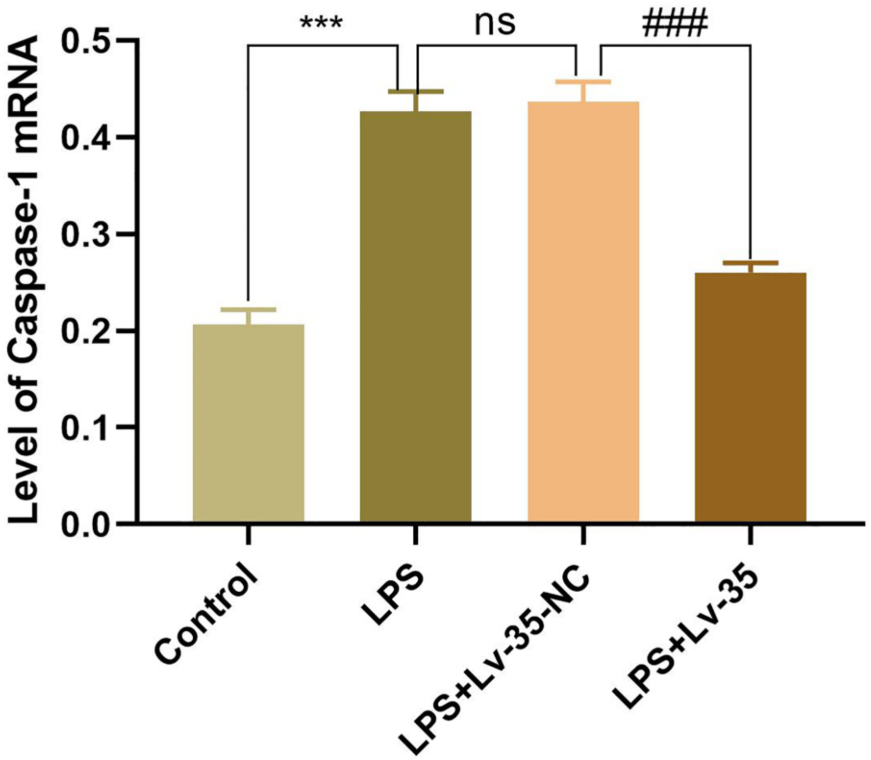

Supplementary Figure 8. The mRNA expression level of Caspase-1. The mRNA expression level of Caspase-1 in LPS group was higher than that in control group, and the difference was statistically significant ($^{***}p < 0.005$). The mRNA expression level of Caspase-1 in LPS+Lv-Hsp22 group was lower than that in LPS+Lv-Hsp22-NC group, and the difference was statistically significant ($^{###}p < 0.005$).

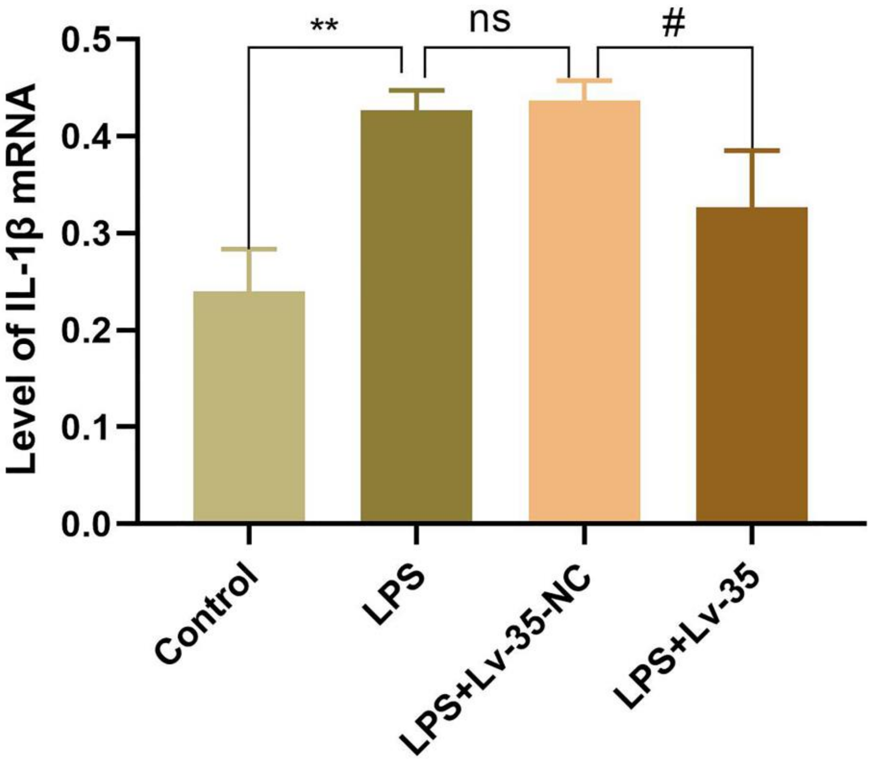

Supplementary Figure 9. The mRNA expression level of IL-1β. The mRNA expression level of IL-1β in LPS group was higher than that in control group, and the difference was statistically significant ($^{**}p < 0.005$). The mRNA expression level of IL-1β in LPS+Lv-Hsp22 group was lower than that in LPS+Lv-Hsp22-NC group, and the difference was statistically significant ($^{\#}p < 0.05$).

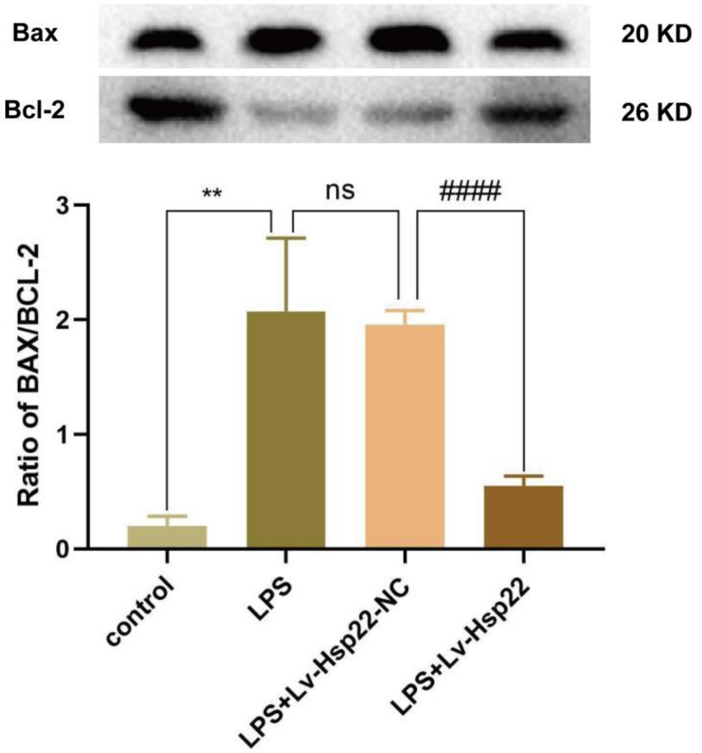

Supplementary Figure 10. The Bax/Bcl-2 ratio *in vivo* experiments. The Bax/Bcl-2 ratio *in vivo* experiments of LPS group was higher than that in control group, and the difference was statistically significant ($^{**}p < 0.005$). The Bax/Bcl-2 ratio *in vivo* experiments of LPS+Lv-Hsp22 group was lower than that in LPS+Lv-Hsp22-NC group, and the difference was statistically significant ($^{####}p < 0.005$).

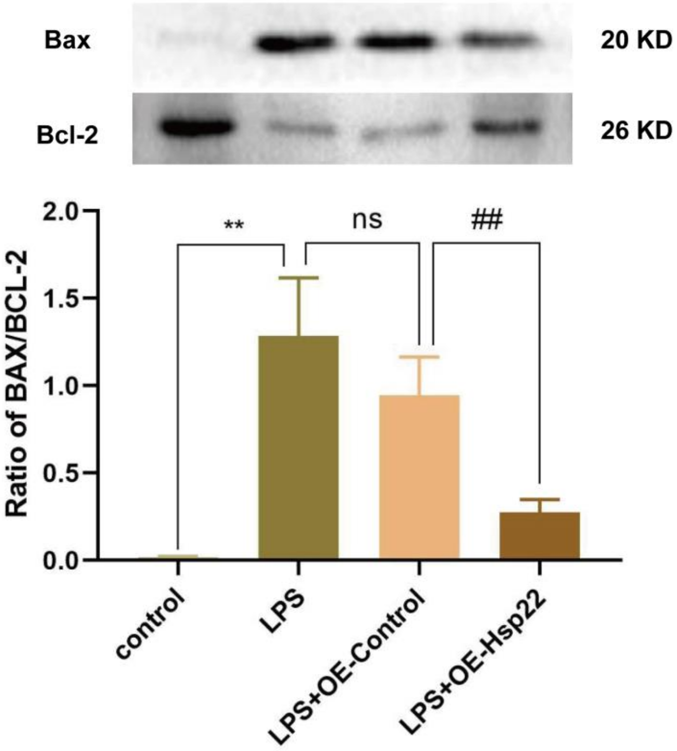

Supplementary Figure 11. The Bax/Bcl-2 ratio *in vitro* experiments. The Bax/Bcl-2 ratio *in vitro* experiments of LPS group was higher than that in control group, and the difference was statistically significant ($^{**}p < 0.005$). The Bax/Bcl-2 ratio *in vivo* experiments of LPS+OE-Hsp22 group was lower than that in LPS+OE-control group, and the difference was statistically significant ($^{##}p < 0.005$).
